# Adipose tissue dysfunction and the liver: reframing MASH as the hepatic expression of systemic metabolic dysfunction

**DOI:** 10.3389/fendo.2026.1920879

**Published:** 2026-10-22

**Authors:** Salvatore Corrao, Christiano Argano

**Affiliations:** 1 Department of Internal Medicine, National Relevance and High Specialization Hospital Trust ARNAS Civico, Di Cristina, Benfratelli, Palermo, Italy; 2 Department of Health Promotion Sciences, Maternal and Infant Care, Internal Medicine and Medical Specialties [PROMISE], University of Palermo, Palermo, Italy

**Keywords:** adipokines, adipose tissue, adipose tissue dysfunction, insulin resistance, lipotoxicity, metabolic dysfunction-associated steatohepatitis, metabolic inflexibility, type 2 diabetes

## Abstract

Metabolic dysfunction-associated steatohepatitis (MASH) is conventionally investigated and treated as a primary disease of the liver. In type 2 diabetes, however, the hepatic lesion is best understood as the visible endpoint of a systemic disorder of adipose tissue. This review develops the mechanistic case for that reading. We trace a single causal narrative: from adipose tissue dysfunction (ATD), the immuno-inflammatory and endocrine failure of dysfunctional adipose tissue, through a reconfigured adipokine secretome and adipose inflammation, to selective insulin resistance and a systemic loss of metabolic flexibility, and thence to the lipid and inflammatory burden that the liver, by virtue of its portal anatomy and metabolic role, is uniquely exposed to. Within this framework, hepatic steatosis is the expected consequence of failed peripheral lipid buffering, and steatohepatitis the readout of lipotoxic, immunometabolic injury rather than an autonomous hepatic process. We argue that MASH is more faithfully interpreted as the hepatic expression of systemic adipose tissue dysfunction than as a primary liver disease, and that this reframing relocates both mechanistic enquiry and the most rational point of therapeutic intervention upstream, to adipose biology and fuel handling. We propose adipose dysfunction as a major, phenotype-dependent upstream driver of T2D-associated MASH that interacts bidirectionally with hepatic, genetic and gut-derived mechanisms, rather than as its sole, universal or unidirectional cause. The argument is advanced as a biologically coherent framework intended to inform how the disorder is conceived and studied.

## 1 Introduction

Metabolic dysfunction-associated steatohepatitis (MASH) is conventionally approached as a disease of the liver. The accumulation of fat within the hepatocyte, its inflammatory injury and its progression towards fibrosis are described, investigated and increasingly treated as though the liver were the seat of the disorder. The recent renaming of the condition as metabolic dysfunction-associated steatotic liver disease, and of its inflammatory form as MASH, was intended partly to correct this impression by foregrounding the metabolic context in which the hepatic lesion arises (1). Yet much of the clinical and experimental literature continues to read the liver as the primary organ of disease, and in type 2 diabetes, where the condition is most prevalent and most aggressive, this hepatocentric framing is particularly entrenched (2).

The idea that MASLD/MASH is a systemic metabolic disease rather than a condition confined to the liver has already been developed, describing non-alcoholic fatty liver disease as a “multisystem disease” and in more recent syntheses of the heterogeneous, metabolism-based pathophysiological mechanism of MASLD (3, 4). The contribution intended here is to organize this systemic character into a single, explicit causal narrative (from ATD, through adipokine and immune dysregulation, to selective insulin resistance and metabolic inflexibility, and thence to hepatic lipid overload and injury) and to identify adipose tissue dysfunction specifically, rather than metabolic disease in a more general sense, as the operative upstream lesion. Because MASLD/MASH is already recognised as a multisystem metabolic disorder, the novelty claimed here lies specifically in integrating adipose dysfunction, adipokine dysregulation, insulin resistance and impaired fuel handling into a single, explicit, adipose-centred mechanistic framework for T2D-associated MASH, rather than in the systemic reading itself. To this end we also introduce an interpretative construct, dysfunctional fat immuno-inflammatory disease (DFIID), intended to unify the endocrine, immune and lipid-handling failures of diseased adipose tissue under a single organ-level framework.

This article argues for a different reading. We propose that MASH in type 2 diabetes is, in a substantial proportion of patients, more faithfully understood as the hepatic expression of a systemic disorder of adipose tissue than as a primary disease of the liver (**Table 1**). It should be acknowledged that contemporary MASLD/MASH models already recognise systemic metabolic mechanisms; **Table 1** contrasts a predominantly liver-focused clinical framing, still common in practice, with the more explicitly adipose-centred reading developed here, rather than implying that current models are unaware of systemic contributors.

**Table 1:** Reframing MASH in type 2 diabetes: from a hepatocentric to an adipose-centred interpretation.

Dimension	Predominantly liver-focused clinical framing	Adipose-centred interpretation
Primary disease origin	Liver	Dysfunctional adipose tissue
Pathophysiological trigger	Hepatic fat accumulation	Adipose tissue dysfunction and impaired adipose expandability
Key biological abnormality	Steatosis	Immunometabolic adipose dysfunction
Central endocrine disturbance	Hepatic metabolic dysregulation	Adipokine network dysfunction
Role of insulin resistance	Consequence of liver disease	Major mediator linking ATD to hepatic injury
Role of metabolic inflexibility	Secondary metabolic disturbance	Functional expression of ATD
Source of hepatic lipid overload	Predominantly hepatic process	Excess adipose-derived lipid flux and *de novo* lipogenesis
Meaning of steatosis	Hepatic pathology	Marker of failed systemic lipid buffering
Meaning of MASH	Progressive liver disease	Hepatic expression of systemic metabolic dysfunction
Main determinant of progression	Liver-centred mechanisms	Severity of adipose dysfunction and systemic metabolic injury
Therapeutic focus	Liver-directed approaches	Upstream restoration of adipose and metabolic health
Conceptual view of the liver	Disease originator	Vulnerable target organ and biological reporter

On this view the hepatocyte is not the origin of the lesion but its most conspicuous reporter: the organ in which a body-wide failure of energy storage and fuel handling becomes histologically visible. The distinction is more than terminological. If the liver is the originator, mechanistic and therapeutic attention belongs there; if it is the reporter, the more rational target lies upstream, in the dysfunctional adipose tissue from which the injurious flux arises (3). This relationship is better characterised as adipose-centred but bidirectional than as strictly one-way: the liver is not a merely passive reporter, since hepatic dysfunction can itself worsen systemic insulin resistance.

Three considerations motivate the reframing. First, hepatic steatosis is so common in type 2 diabetes as to be almost the norm, yet only a minority of steatotic livers progress to steatohepatitis and its sequelae; the presence of fat is therefore a poor predictor of disease, and what distinguishes the progressors is not the quantity of lipid but the metabolic context in which it accumulates (2). Second, that context is systemic: the determinants of progression such as the severity of adipose dysfunction, the intensity of the lipotoxic flux, the degree of insulin resistance, are properties of the organism rather than of the liver alone. Third, the clinical burden of the condition extends well beyond the liver, a fact that is difficult to reconcile with a purely hepatic account but follows naturally from a systemic one (4). Taken together, these observations point away from the liver as the primary lesion and towards the tissue whose dysfunction sets the whole process in motion.

The purpose of this review is to develop that systemic, adipose-centred account in mechanistic detail, and to do so as a self-contained argument, tracing the causal sequence outlined above through to its hepatic consequences (**Figure 1**).

**Figure 1:**
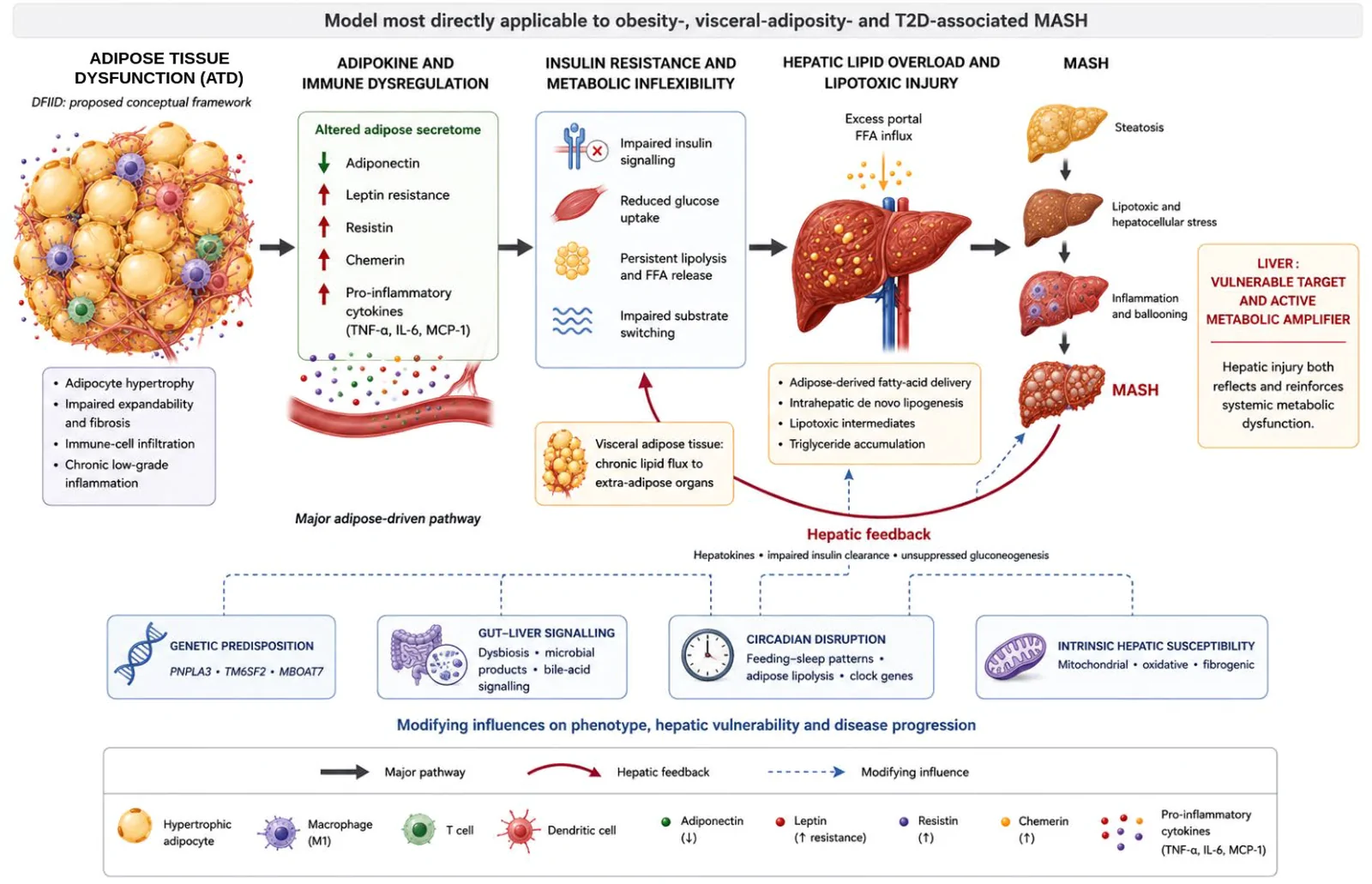
Adipose tissue dysfunction as the upstream driver of MASH in type 2 diabetes. Proposed mechanistic framework linking dysfunctional adipose tissue to the development of metabolic dysfunction-associated steatohepatitis (MASH) in type 2 diabetes. The sequence begins with adipose tissue dysfunction (ATD), defined as the immuno-inflammatory and endocrine dysfunction of adipose tissue characterised by impaired adipose expandability, adipocyte hypertrophy, tissue fibrosis, immune-cell infiltration and chronic low-grade inflammation. Adipose dysfunction is accompanied by a reconfiguration of the adipokine network, including reduced adiponectin signalling and increased pro-inflammatory mediators, which contribute to systemic insulin resistance and impaired metabolic regulation. The resulting insulin-resistant state is associated with metabolic inflexibility, whereby the adaptive switching between glucose and fatty-acid utilisation is progressively lost. Persistent lipolysis, increased free fatty-acid flux and impaired substrate handling promote hepatic lipid overload through both enhanced fatty-acid delivery and increased *de novo* lipogenesis. When the hepatocyte’s capacity for safe lipid storage and disposal is exceeded, lipotoxic stress, inflammation and hepatocellular injury develop, driving progression from simple steatosis to MASH. Within this framework, the liver is interpreted as a major downstream site of systemic adipose tissue dysfunction rather than the primary origin of disease, but not as a passive one: a feedback arrow indicates how hepatic dysfunction itself worsens systemic insulin resistance, and dashed arrows indicate modifying influences, genetic predisposition, gut-liver signalling, circadian disruption and intrinsic hepatic susceptibility, that shape phenotype and severity rather than acting through a single linear pathway. The figure is intended as an interpretative model illustrating the biological continuity between ATD, insulin resistance, metabolic inflexibility and hepatic injury in type 2 diabetes. IL-6, interleukin-6; MCP-1, monocyte chemoattractant protein-1; MASH, metabolic dysfunction-associated steatohepatitis; TNF-α, tumour necrosis factor-α.

We confine the discussion deliberately to the adipose–liver axis and to the biology that links the two, and we frame the resulting model not as established doctrine but as a biologically coherent interpretation intended to inform how MASH is conceived, investigated and, ultimately, treated. This review is a narrative synthesis rather than a systematic review. Literature was identified through PubMed and Google Scholar for the period January 2000–June 2026, using combinations of the terms “adiposopathy”, “adipose tissue dysfunction”, “adipokine”, “insulin resistance”, “metabolic inflexibility”, “MASLD”, “MASH”, “nonalcoholic steatohepatitis” and “type 2 diabetes”, supplemented by hand-searching the reference lists of key reviews. Evidence was prioritised for mechanistic clarity, direct relevance to the adipose–liver axis in type 2 diabetes, and, where available, human data; competing and modifying mechanisms (genetic, gut-derived, circadian and intrinsic hepatic factors) were deliberately included to represent the field rather than to selectively support the adipose-centred reading.

## 2 Adipose tissue dysfunction as an immunometabolic disorder

The starting point of the argument is the recognition that adipose tissue can be diseased independently of its mass. The concept of adipose tissue dysfunction (ATD) was introduced to capture precisely this: the anatomical and functional derangement of adipose tissue that develops under chronic positive energy balance in susceptible individuals, and that drives cardiometabolic risk more faithfully than adiposity per se (5). The clinical observations that the concept was designed to explain are familiar: the individual with substantial fat mass who remains metabolically healthy, and the lean individual who nonetheless carries disproportionate metabolic risk, and they make the same essential point: it is the quality of adipose tissue, not its quantity, that determines metabolic harm (6). ATD thus reframes obesity-associated disease as a disorder of adipose function rather than of fat accumulation, and in doing so identifies adipose tissue as an organ that can fail. Central to this failure is the limited capacity of subcutaneous adipose tissue to expand safely. In health, the subcutaneous depot behaves as a metabolic buffer, accommodating surplus energy through adipocyte hyperplasia and hypertrophy and thereby protecting other tissues from lipid exposure. The adipose expandability hypothesis holds that metabolic disease emerges when this buffering capacity is exceeded: once the subcutaneous depot can no longer safely store incoming lipid, the surplus is diverted to visceral and ectopic sites that tolerate it poorly (7). The failing depot is itself pathological (marked by adipocyte hypertrophy, local hypoxia, cell death, extracellular-matrix remodelling and fibrosis) so that its storage capacity tends to fail at precisely the moment of greatest demand, accelerating the spillover of lipid into the liver, skeletal muscle, pancreas and beyond (8).

Adipose tissue is, moreover, a highly plastic organ rather than a static store. Its expansion in visceral obesity involves coordinated adipocyte hypertrophy and hyperplasia, angiogenic remodelling of the microvasculature, extracellular-matrix turnover and dynamic changes in the resident immune-cell population, and it is the failure of this coordinated remodelling, rather than lipid accumulation as such, that converts an expanding depot into a dysfunctional one; when expansion outstrips its vascular supply, local hypoxia and oxidative stress ensue and the secretory output of the tissue is reconfigured accordingly (9). Cellular quality control forms part of this plasticity. Autophagy, including its lipid- and mitochondria-selective forms lipophagy and mitophagy, governs adipocyte differentiation, lipid-droplet turnover and organelle integrity, and its dysregulation under nutrient excess contributes to adipocyte stress, inflammasome activation and impaired insulin signalling, thereby linking a housekeeping process to the organ-level failure described here (10).

The anatomy of fat distribution makes this spillover especially consequential for the liver. Visceral adipose tissue is both more prone to dysfunction than the subcutaneous depot and more hazardous in its consequences, in part because its venous drainage enters the portal circulation directly (8). Free fatty acids and adipocyte-derived mediators released from a dysfunctional visceral depot therefore reach the liver in concentrated form, before systemic dilution, exposing the hepatocyte to the products of adipose failure ahead of most other tissues. This portal relationship is one reason why central, rather than generalised, adiposity is the adipose phenotype most closely associated with steatotic liver disease, and it foreshadows the liver’s particular vulnerability (11).

The relevant anatomy is not exhausted by the visceral-subcutaneous dichotomy. Adipose tissue also accumulates at intermuscular, epicardial, perivascular, perirenal and pancreatic sites, each with distinct cellular origins, secretory profiles and metabolic consequences. Intermuscular adipose tissue is quantitatively comparable to the visceral depot in some individuals, is as lipolytic as visceral fat and around three times more lipolytic than subcutaneous fat, secretes a distinctly inflammatory and immunogenic secretome, and is independently associated with insulin resistance and type 2 diabetes (12). Because these depots differ in the magnitude and composition of the lipid and cytokine flux they generate, and because only the visceral depot drains directly into the portal vein, the hepatic consequences of a given total fat mass depend substantially on where that fat is located and on how dysfunctional it is. MASH is therefore more faithfully understood as the hepatic outcome of a particular distribution of dysfunctional adipose tissue than of adiposity in aggregate.

That adipose dysfunction, rather than fat mass, is the operative variable is underlined by the wide variation between individuals in the amount of lipid they can store before metabolic disease appears. The notion of a personal fat threshold captures this idea: each individual appears to have a limit to safe adipose expansion, above which lipid overflows to ectopic sites, and that limit varies considerably and is in part genetically determined (7). The extreme case of lipodystrophy, in which the near-absence of functional adipose tissue produces severe insulin resistance and florid hepatic steatosis despite minimal fat mass, is the clearest demonstration that it is the capacity of adipose tissue to store lipid safely, and not the quantity of lipid as such, that protects the liver (3). ATD and lipodystrophy may thus be seen as the two ends of a single axis of adipose failure, both arriving, by opposite routes, at a liver exposed to lipid it cannot accommodate.

This inter-individual variation has been formalised into a set of recognised obesity sub-phenotypes that differ materially in hepatic risk. Metabolically healthy obesity, metabolically unhealthy non-obesity (encompassing normal-weight obesity and the metabolically obese normal-weight phenotype), metabolically unhealthy obesity, its sarcopenic-obesity subtype, and the lipodystrophic phenotypes are distinguished not by body mass index but by body composition and metabolic status, and they carry graded risks of insulin resistance, type 2 diabetes and steatotic liver disease (13). Phenotypes characterised by ectopic fat deposition and reduced appendicular skeletal muscle mass, including normal-weight obesity and sarcopenic obesity, may be associated with hepatic steatosis despite an unremarkable body mass index, whereas metabolically healthy obesity carries a comparatively lower, though not absent, hepatic risk and is in any case frequently transient. This gradient is difficult to reconcile with a model in which hepatic fat is the primary lesion, but follows directly from one in which the liver reports the functional state of adipose tissue.

Upstream of ATD itself lie the exposures that generate it. Chronic positive energy balance, and in particular the obesogenic dietary environment of high-income and rapidly transitioning societies, is the principal proximate cause of the adipose expansion that eventually exceeds an individual’s safe storage capacity. Ultra-processed foods are central to this environment. In a randomised inpatient crossover trial in which ultra-processed and unprocessed diets were matched for presented calories, energy density, macronutrients, sugar, sodium and fibre, participants eating ad libitum spontaneously consumed approximately 500 kcal per day more, and gained weight, on the ultra-processed diet, showing that an ultra-processed dietary pattern can promote excess energy intake even when several presented nutritional variables are matched (14). Prospective and mechanistic data extend this to the liver: higher ultra-processed food consumption is associated with an increased risk of incident MASLD, an association only partly mediated by adiposity, and the mechanisms invoked, emulsifier- and additive-induced alterations of the gut microbiota and intestinal barrier, a high glycaemic and fructose load favouring *de novo* lipogenesis, and advanced glycation end-products promoting oxidative stress and inflammation, converge on precisely the adipose and hepatic pathways described in this review (15).

Obesity and obesogenic diets are therefore best regarded as upstream drivers of ATD rather than as parallel or competing explanations. They act by imposing a sustained lipid load on a depot of finite and partly genetically determined capacity; ATD is what that depot does when the load exceeds what it can safely accommodate, and the hepatic lesion is what follows downstream. The same logic accounts for the reverse direction: sustained negative energy balance, whether achieved by dietary restriction, pharmacotherapy or bariatric surgery, relieves the load, improves adipose insulin sensitivity and attenuates adipose inflammation, and is accompanied by regression of hepatic steatosis and steatohepatitis. It also explains why hepatic steatosis and insulin resistance are commonly demonstrable in individuals with obesity well before type 2 diabetes becomes clinically manifest: the adipose lesion, and its hepatic consequence, precede the glycaemic one.

We find it useful to conceive of pathogenic adipose tissue as a dysfunctional fat immuno-inflammatory disease (DFIID), an interpretative construct (not a defined nosological entity) intended to capture the convergence of endocrine, immune and lipid-handling failure that characterises diseased fat. DFIID is proposed purely as a conceptual, organ-level construct to guide interpretation and research, and not as an established diagnostic or clinical entity; it carries no diagnostic criteria and is not intended for clinical staging, coding or individual patient classification. Its added value over the term “adipose tissue dysfunction” alone is organisational rather than diagnostic: DFIID explicitly names the immune and inflammatory component of adipose failure alongside the endocrine one, providing a single label under which the adipokine, immune-cell and lipid-handling abnormalities can be grouped for the purposes of this argument; it is not proposed to supersede adipose tissue dysfunction as a research or clinical term.

The value of such a framing is that it resists reducing ATD to any single abnormality, whether of a hormone, a cytokine or a lipid species, and instead emphasises the coordinated failure of adipose tissue as an organ. It is this organ-level failure, rather than excess adiposity in the abstract, that initiates the cascade culminating in hepatic disease, and the sections that follow examine its two principal outputs: a reconfigured adipokine secretome with sustained inflammation, and a body-wide disturbance of insulin action and fuel handling.

It should be stated clearly that ATD is presented here as a major, upstream contributor to MASH rather than as its sole or universal cause. MASLD/MASH is increasingly recognised as a multifactorial disorder involving adipose dysfunction, genetic predisposition (including common variants in PNPLA3, TM6SF2 and MBOAT7), alterations to hepatocyte lipid and carbohydrate metabolism, mitochondrial dysfunction, dysregulated bile acid signalling through the farnesoid X receptor and TGR5, immune system dysregulation, oxidative stress and signals from the gut reaching the liver via the gut-liver axis (3, 4) These mechanisms do not replace the adipose-centred account presented here, but rather act alongside it. In some cases, they amplify the flux of adipose-derived lipids and inflammation, while in others, they modify individual susceptibility independently.

## 3 Dysfunctional adipokine networks and adipose inflammation

The first output of adipose failure is immunological. More than two decades ago, human and experimental studies established that obese adipose tissue is infiltrated by macrophages in proportion to adipocyte size and body mass, and that these cells are a principal local source of pro-inflammatory cytokines (16, 17). Subsequent work showed that obesity does not merely recruit macrophages but alters their character, favouring a classically activated, pro-inflammatory phenotype that aggregates in crown-like structures around dying adipocytes, at the expense of the alternatively activated, tissue-reparative population that predominates in healthy fat (18). This polarised, inflamed milieu is sustained by mediators such as tumour necrosis factor-α, interleukin-6 and monocyte chemoattractant protein-1, and chemokine-driven recruitment of monocytes is sufficient, in experimental systems, to provoke both local and systemic insulin resistance and to promote hepatic lipid accumulation (19). Adipose tissue, on this evidence, is not an inert store but an active immunometabolic organ whose inflammatory tone helps set the metabolic state of the whole body (20).

The M1/M2 dichotomy used here is a convenient simplification of a more graded reality. Following the classification summarised by Yao et al., alternatively activated macrophages can be further described as M2a, M2b, M2c and M2d subtypes according to context, polarising stimuli and functional profile. M2a macrophages are typically induced by interleukin-4 and interleukin-13 and are associated with tissue repair and wound healing; M2b macrophages are linked to interleukin-1/Toll-like receptor signalling and an immunoregulatory cytokine profile; M2c macrophages are induced by interleukin-10, transforming growth factor-β and glucocorticoids and participate in clearance of apoptotic cells and tissue remodelling; and M2d macrophages exhibit an angiogenic phenotype characterised by vascular endothelial growth factor production (21). This finer classification underscores the plasticity of adipose-tissue macrophages and the limitations of a binary M1/M2 description: their phenotype is shaped by the local metabolic and immune microenvironment and can shift as adipose tissue expands and becomes dysfunctional. Two terms are useful in specifying what is meant by inflammation in this setting, and they map onto the two steps of the argument. Adipoflammation denotes the chronic, sterile inflammatory transformation of the visceral depot itself: adipocyte hypertrophy and lipid overload, tissue hypoxia, infiltration by monocytes and macrophages through the Toll-like receptor 4-MCP-1/CCL2 axis, a shift from anti-inflammatory (adiponectin, CTRP-3) towards pro-inflammatory (leptin, resistin) adipokines, replacement of interleukin-4- and interleukin-13-responsive M2 macrophages by lipopolysaccharide- and interferon-γ-responsive M1 macrophages, and the formation of crown-like structures around necrotic adipocytes, with a consequent fall in adipocyte insulin sensitivity and a rise in lipolysis (22). Metaflammation denotes its systemic sequel: the low-grade, chronic elevation of C-reactive protein, tumour necrosis factor, interleukin-1 and interleukin-6 that spills over from the inflamed depot into the circulation, impairs insulin signalling through cytokine-induced inhibitory phosphorylation of the insulin receptor and insulin receptor substrate-1, and is accompanied by ectopic lipid deposition in liver, muscle, heart and pancreas (22). The distinction matters for the account developed here because it names the two stages by which a local adipose lesion becomes a systemic and ultimately hepatic one: adipoflammation first, metaflammation second, with the liver positioned at the receiving end of both. It also makes explicit why adipose tissue, which synthesises cytokines, chemokines, complement factors, Toll-like receptors, antimicrobial peptides and the inflammasome complex, is properly regarded as a component of the innate immune system rather than as an inert store (22). This is precisely the organ-level immunometabolic failure that the DFIID construct introduced above is intended to capture, and the terminology of adipoflammation and metaflammation provides the mechanistic vocabulary in which that construct can be expressed.

Importantly, the relationship between inflammation and metabolic dysfunction is bidirectional rather than linear. Inflammation impairs insulin signalling, but insulin resistance can itself initiate and sustain adipose inflammation, so that immune activation is at once a cause and a consequence of the metabolic lesion (23). This circularity matters for the present argument because it means that adipose inflammation, once established, becomes self-reinforcing: the inflamed depot is less able to store lipid safely, the resulting ectopic lipid flux aggravates insulin resistance, and the heightened insulin resistance feeds back upon the adipose tissue to perpetuate the inflammatory state. The adipose organ thus tends towards a stable pathological equilibrium rather than spontaneous resolution.

The second, and complementary, output is endocrine. Diseased adipose tissue communicates with distant organs through a profoundly altered secretome. Adiponectin sits at the centre of this network as its principal protective signal: insulin-sensitising, anti-inflammatory and hepatoprotective, it acts through its receptors to promote fatty acid oxidation and restrain hepatic lipogenesis, and its circulating concentration falls as adipose dysfunction advances (24). The decline of adiponectin is arguably the single most consistent endocrine marker of adipose failure, and its loss removes a key brake on hepatic lipid accumulation and inflammation (25). Against this diminishing protective tone, leptin rises with fat mass yet loses much of its central metabolic efficacy in the obese, insulin-resistant state, while contributing peripherally to a pro-inflammatory and pro-fibrotic milieu; resistin and other mediators associate further with insulin resistance and low-grade inflammation (25, 26).

A further and until recently underappreciated endocrine input to adipose tissue is provided by bile acids. Beyond their familiar enterohepatic and luminal roles, bile acids circulating in the systemic, non-portal compartment act on white adipocytes as steroidal hormones through the nuclear receptor FXR and the membrane receptor TGR5, regulating adipocyte differentiation, lipid accumulation, hypoxia, autophagy, insulin signalling, glucose uptake and adipokine and cytokine secretion; the term “bilokines” has been proposed for this specific receptor interaction in adipocytes (22). Systemic bile-acid signalling therefore constitutes a route by which intestinal and hepatic signals feed back onto adipose tissue, and a plausible contributor to the rapid improvement in adipose and systemic insulin sensitivity that follows bariatric surgery, before and out of proportion to the weight lost. Its inclusion reinforces the bidirectional reading adopted throughout: adipose tissue is the upstream lesion of the sequence described here, but it is not insulated from the hepatic and gut-derived signals that return to it.

The salient abnormality, then, is not the perturbation of any single adipokine but a coordinated reconfiguration of the network, in which the loss of adiponectin-mediated restraint permits the pro-inflammatory and lipogenic signals of leptin, resistin and the cytokine milieu to predominate. Through this reconfigured secretome, the structural and immune failure of adipose tissue is translated into a circulating signal that the liver and other insulin-responsive tissues cannot ignore. The adipose organ, in other words, does not merely fail to store lipid; it actively broadcasts its dysfunction, and it is to the systemic consequences of that broadcast, disordered insulin action and the loss of metabolic flexibility, that we now turn.

## 4 Insulin resistance and metabolic inflexibility

The combined lipid and inflammatory burden emanating from dysfunctional adipose tissue converges on insulin action. Insulin resistance is often portrayed as a uniform loss of insulin’s effects, but the hepatic phenotype reveals a more specific and more revealing abnormality. In the insulin-resistant liver, insulin fails to suppress gluconeogenesis yet continues to drive lipogenesis, so that elevated glucose output and accelerated fat synthesis coexist: the paradox of selective, or pathway-selective, insulin resistance (27, 28). The lipogenic branch of insulin signalling remains responsive even as its glucoregulatory branch fails, with the consequence that the compensatory hyperinsulinaemia provoked by peripheral resistance does not restrain hepatic fat synthesis but amplifies it. Hyperinsulinaemia is, in this sense, as much a driver of hepatic lipid accumulation as a marker of disordered glucose homeostasis.

The mechanistic basis of this selectivity has been clarified by work implicating lipid intermediates in the impairment of insulin signalling. The accumulation of diacylglycerols within the hepatocyte activates protein kinase C-ϵ, which attenuates insulin-receptor signalling towards glucose metabolism while leaving lipogenic transcriptional programmes intact; ceramides contribute through parallel pathways (29). The continuous delivery of free fatty acids from adipose tissue that can no longer buffer them, together with the substrate provided by hyperinsulinaemia-driven *de novo* lipogenesis, supplies precisely the lipid species that perpetuate this selective resistance. Skeletal-muscle insulin resistance compounds the problem at the whole-body level, diverting ingested carbohydrate away from peripheral oxidation and towards the liver, where it furnishes further substrate for lipid synthesis.

These disturbances are most coherently understood through the concept of metabolic flexibility: the capacity of healthy tissues to switch between glucose and fatty acid oxidation according to nutritional state, oxidising glucose after a meal and fatty acids during fasting. The principle has a long lineage, reaching back to the description of the glucose–fatty acid cycle, and was later characterised in human skeletal muscle as a measurable blunting of substrate switching in the insulin-resistant state (30, 31). Metabolic inflexibility, the failure of this adaptive switching, is now recognised as a systemic feature of insulin-resistant metabolism rather than a property of muscle alone (32). When the fasting-to-feeding transition fails, the delivery of fatty acids to liver and muscle becomes continuous rather than episodic; lipid that can be neither buffered by adipose tissue nor oxidised on demand accumulates ectopically, is esterified, or is increasingly synthesised *de novo* within the hepatocyte (33).

It is worth stating plainly that metabolic flexibility resists precise measurement, and that under clamp conditions much of the apparent defect reflects the rate of glucose disposal rather than an independent failure of substrate switching (33). We therefore treat metabolic inflexibility not as a validated biomarker but as an integrative description of the disordered fuel economy that adipose failure imposes: a state in which the supply of lipid to oxidative tissues is no longer matched to demand. Conceived in this way, the sequence from adipose dysfunction to disordered fuel handling is continuous: sick fat releases lipid it can no longer store, insulin resistance directs that lipid towards synthesis and ectopic deposition rather than disposal, and the loss of flexible substrate switching ensures that the surplus persists. The organ on which this surplus is most heavily and most visibly deposited is the liver.

An additional layer of regulation is the circadian organisation of hepatic metabolism. Under physiological conditions, core clock genes impose a temporal structure on hepatic bioenergetics, coordinating the timing of lipogenesis, gluconeogenesis and mitochondrial fatty acid oxidation with the fasting–feeding cycle, so that metabolic flexibility is not only a matter of substrate availability but also of when the hepatocyte is competent to switch between substrates (34). Disruption of this circadian architecture (whether through irregular feeding patterns, sleep disturbance or the metabolic disease process itself) reduces the normal oscillation of lipogenic and oxidative gene programmes. Adipose tissue itself is subject to comparable clock control: disrupted circadian signalling blunts the normal diurnal rhythm of adipocyte lipolysis and insulin sensitivity, so that free fatty-acid flux to the liver becomes less predictable and less well matched to hepatic oxidative capacity, compounding the substrate-flux disturbance described above. This means that the liver’s capacity to buffer a lipid load becomes less of a fixed property of the organ, and more dependent on the time of day at which the load is delivered. Incorporating this circadian dimension introduces an additional source of heterogeneity, both between and within individuals: two patients with comparable adipose dysfunction may have different hepatic outcomes depending on the regularity of their feeding and sleep schedules. Similarly, the same patient may be more or less vulnerable to a given lipid flux depending on when it occurs. While this perspective does not alter the central adipose-driven argument presented here, it does refine it by suggesting that chronotherapeutic strategies aligned with the hepatocyte’s endogenous clock could complement interventions directed at adipose tissue itself.

## 5 The liver as a vulnerable target organ

That the liver should be the first and most affected recipient of adipose failure is not accidental but follows from its anatomy and its metabolic role. The liver is the principal hub of intermediary metabolism, positioned to receive nutrients absorbed from the gut and mediators draining from the visceral adipose depot through the portal vein, and equipped to store, oxidise, synthesise and export lipid in response to systemic demand (35). This central position, which makes the liver indispensable to whole-body fuel homeostasis in health, becomes a liability when the signals it integrates are pathological. The hepatocyte is exposed, before most other tissues, to the concentrated free fatty acid flux and adipokine signature of a dysfunctional visceral depot, and it responds to that exposure with the metabolic adjustments for which it is designed, adjustments that, under sustained overload, become injurious.

The sources of hepatic lipid in this state are well defined. Hepatic triglyceride accumulation reflects both adipose-derived fatty-acid delivery and intrahepatic *de novo* lipogenesis driven by systemic metabolic dysregulation, rather than a predominantly extrahepatic process. Isotopic studies in human fatty liver attribute the largest single fraction of stored hepatic triglyceride to circulating fatty acids derived from adipose lipolysis, with *de novo* lipogenesis, itself upregulated by the hyperinsulinaemia of systemic insulin resistance, contributing a disproportionately large and pathologically expanded share, and dietary fat a smaller fraction (36). The dominance of adipose-derived and lipogenic fatty acids is itself an argument for the systemic reading advanced here: the lipid that fills the hepatocyte is, for the most part, not generated by an autonomous hepatic defect but delivered to, and synthesised within, the liver as a consequence of adipose failure and the hyperinsulinaemia that accompanies it. The liver accumulates fat because it is the organ to which the surplus is consigned.

Crucially, the threat to the hepatocyte is not posed by stored triglyceride itself, which is comparatively inert and may even be protective as a means of sequestering reactive lipid. The danger lies in the lipotoxic intermediates that accumulate when the influx of fatty acids exceeds the hepatocyte’s capacity to store or safely oxidise them: diacylglycerols, ceramides and related species that disrupt insulin signalling, perturb membranes and organelles, and trigger cellular stress (37, 38). The mitochondrion is central to this transition: overloaded with substrate and unable to dispose of it through complete oxidation, it generates reactive oxygen species and incompletely oxidised lipid, while mitochondrial and endoplasmic-reticulum stress amplify the injurious signal (39, 40). Hepatic steatosis is therefore best understood as the point at which the liver’s buffering capacity is first exceeded, and lipotoxicity as the consequence of exceeding it.

The liver does not accept this surplus passively. It attempts to dispose of the incoming lipid by exporting it as very-low-density lipoprotein and by increasing fatty acid oxidation, and for a time these compensatory routes limit intrahepatic accumulation (35). Their capacity is finite, however, and is itself constrained by the same insulin-resistant, hyperinsulinaemic state that drives the lipid influx, so that export and oxidation are eventually outpaced by supply. When that happens, lipid accrues within the hepatocyte not as a sign of any hepatic appetite for fat but as evidence that the organ’s disposal mechanisms have been overwhelmed. The steatotic liver is, in this sense, a saturated buffer: a tissue continuing to perform its physiological role of lipid handling under a load that exceeds its capacity, with lipotoxic injury the price of that saturation.

Read in this way, the liver is a vulnerable target rather than a primary actor. Its vulnerability is structural, a function of portal exposure and metabolic centrality, and its injury is imposed by a flux it did not generate. This does not diminish the importance of hepatic events, which are real and clinically consequential, but it relocates their cause. The hepatocyte’s stress is the local manifestation of a systemic failure of lipid storage and fuel handling, and the progression from simple steatosis to steatohepatitis, considered next, is the unfolding of that stress within the liver.

The relationship between diabetes and liver disease is better characterised as bidirectional than as a one-way consequence of adipose failure alone. Type 2 diabetes and its associated hyperinsulinaemia accelerate hepatic lipid accumulation and fibrogenesis via the aforementioned mechanisms. Conversely, the fatty and inflamed liver itself contributes significantly to whole-body glucose dysregulation through impaired hepatic insulin clearance, increased gluconeogenesis that is difficult to suppress, and the release of pro-atherogenic and pro-diabetogenic hepatokines such as fetuin-A and selenoprotein P (41). These hepatic signals affect skeletal muscle and pancreatic β-cell function, meaning that hepatic steatosis is an active participant in the progression of diabetes, not merely a marker of its severity. This circuitry, in which adipose-driven hepatic injury worsens systemic insulin resistance and β-cell stress, which in turn sustain adipose dysfunction, reinforces the systemic framework proposed here. It identifies the liver as not only a marker of adipose disease, but also a node that can amplify metabolic disturbances, which has direct implications for how the relationship between diabetes and the liver is monitored and treated in clinical practice (42).

## 6 From steatosis to MASH

The transition from steatosis to steatohepatitis is the crux of the disease, because it separates the common and largely benign from the uncommon and consequential. The older conception of this transition, a benign first hit of fat accumulation followed by a discrete inflammatory second hit, has given way to a model of multiple parallel hits, in which numerous insults arising concurrently from a single metabolic source act together on a susceptible liver (43). This shift in understanding is consonant with the systemic account: the insults that drive progression lipotoxic lipid species, oxidative and organelle stress, inflammatory signalling and altered gut-derived signals are not sequential hepatic events but the simultaneous hepatic consequences of sustained adipose-derived overload.

When lipotoxic stress exceeds the hepatocyte’s adaptive capacity, a stereotyped sequence of injury ensues. Endoplasmic-reticulum stress, mitochondrial dysfunction and oxidative stress converge to activate stress-responsive and inflammatory signalling, engage the inflammasome and recruit innate immune cells, so that resident and infiltrating macrophages sustain a pro-inflammatory milieu around injured and dying hepatocytes (40, 44). Recent experimental work supports this idea, showing that markers of oxidative damage are closely linked to the severity of hepatic injury and indices of systemic metabolic disturbance. This suggests that oxidative stress is a key mechanism linking the metabolic overload described here to the resulting hepatocellular injury (45). It is more faithful to describe this as immunometabolic injury than as inflammation alone, because the same coupling of metabolic overload to immune activation that characterises dysfunctional adipose tissue is recapitulated within the liver: the hepatic macrophage responds to lipid and danger signals, and its response in turn aggravates the metabolic lesion. The parallel is not incidental but reflects a shared biology, in which lipid excess and immune activation are inseparable.

Signals arriving from the gut add a further dimension to this hepatic phase. The liver receives, through the portal circulation, microbial products and metabolites whose composition is altered in metabolic disease, and these appear to modulate innate-immune activation and to contribute to the inflammatory tone of the steatotic liver (46). The gut–liver relationship does not displace the adipose-centred account but complements it, supplying an additional source of the danger signals to which the lipid-laden hepatocyte and its associated macrophages respond. Hepatocellular injury, with its characteristic ballooning degeneration and cell death, is the visible expression of these converging stresses (47).

This gut contribution deserves closer specification, since the underlying mechanisms are increasingly well characterised. Chronic overnutrition and adipose dysfunction are associated with intestinal dysbiosis, a shift in microbial community structure that alters the profile of bacterial metabolites, including short-chain fatty acids, secondary bile acids and, notably, lipopolysaccharide, reaching the liver (48). This dysbiosis coincides with impairment of intestinal barrier integrity, allowing increased translocation of microbial products across a more permeable gut epithelium and into the portal circulation (49). Once delivered to the liver, these bacterial metabolites engage pattern-recognition receptors, most notably Toll-like receptor 4, on resident Kupffer cells and infiltrating macrophages, triggering an innate-immune activation that compounds the lipotoxic injury described above and accelerates the transition from steatosis to steatohepatitis (48, 49). The gut–liver axis therefore operates as a second, largely independent route by which a disturbed metabolic and microbial environment reaches the hepatocyte, running in parallel with, rather than substituting for, the adipose-derived lipid and adipokine flux.

Steatohepatitis, on this analysis, is not a disease of fat accumulation but the hepatic readout of lipotoxic, immunometabolic injury, the point at which a systemic failure of lipid storage and fuel handling becomes written into the structure and function of the liver. Persistent injury of this kind elicits a wound-healing response, with activation of hepatic stellate cells and deposition of extracellular matrix, and it is this fibrogenic response that ultimately determines the long-term hepatic consequences of the disorder (47, 50). We do not pursue fibrosis progression and its clinical assessment here, as these lie beyond the scope of the present argument; for the purposes of this review the essential point is that the inflammatory, injurious phase of MASH arises from, and remains continuous with, the systemic adipose biology described in the preceding sections. In the adipose-driven majority of cases, the liver may represent a major downstream site of systemic metabolic injury in adipose-driven MASH phenotypes, rather than an autonomous disease origin.

## 7 Clinical and conceptual implications

If MASH in type 2 diabetes is the hepatic expression of systemic adipose dysfunction, then several consequences follow for how the disorder is conceived and approached. The most immediate is that the rational target of intervention is the upstream biology rather than the hepatic fat itself. Measures that act on adipose function and fuel handling, the reduction of visceral adiposity, the restoration of safe lipid storage and the recovery of metabolic flexibility, address the disorder at its source, whereas measures aimed solely at the liver address a downstream manifestation. This is consistent with the observation that the interventions with the most reliable hepatic benefit are those that act systemically (3).

Weight loss achieved through dietary energy restriction and physical activity remains the foundation of this approach, acting directly on adipose mass, body composition and substrate handling, with hepatic histological improvement scaling with the magnitude of weight reduction. Pharmacological strategies illustrate the same principle from a mechanistic standpoint: thiazolidinedione therapy improves insulin sensitivity and raises adiponectin, and is associated with histological improvement in steatohepatitis, an effect attributable to its action on adipose biology rather than on the hepatocyte directly (50). Incretin-based therapies that reduce adiposity and improve metabolic control have likewise been shown to resolve steatohepatitis, again through amelioration of the upstream metabolic state rather than a primarily hepatic mechanism (51). Where obesity is severe, metabolic surgery produces the most durable reduction in dysfunctional adipose mass and the most consistent hepatic histological improvement, a finding that is difficult to explain on a hepatocentric model but follows naturally from a systemic one (52). We present these observations to illustrate the coherence of the framework rather than to prescribe management, which lies beyond the scope of this article. Consistent histological improvement across mechanistically diverse, adipose-directed interventions supports systemic metabolic involvement in MASH and is compatible with adipose dysfunction as a major upstream driver; it does not, on its own, prove adipose tissue to be the sole origin of the disease, since therapeutic response and causal origin are logically distinct.

Although these therapies are unified by their capacity to relieve adipose dysfunction, they do not all act through identical mechanisms. Distinguishing between systemic and hepatically directed effects strengthens the framework proposed here. Thiazolidinediones and, to a lesser extent, incretin-based agents predominantly act upstream. They improve adipose insulin sensitivity, restore adiponectin signalling, and reduce the peripheral lipolytic and inflammatory flux reaching the liver. This is consistent with the systemic account developed above. However, other agents appear to combine such systemic metabolic benefits with direct hepatic action. For example, glucagon-like peptide-1 and glucose-dependent insulinotropic polypeptide receptor co-agonism has been associated with reductions in hepatic *de novo* lipogenesis and improved hepatocellular fatty acid oxidation that exceed what would be predicted from weight loss alone. Furthermore, fibroblast growth factor 21 analogues act directly on the hepatocyte to suppress lipogenesis and promote fatty acid oxidation, independently of changes in adipose mass (7).

Recognising this distinction is important both clinically and conceptually. Clinically, agents with genuine direct hepatic action may retain efficacy even when the systemic, adipose-directed response is incomplete. Conceptually, it indicates that, while the liver is not the originator of the disorder, it is not an entirely passive bystander to its treatment either. The conceptual implications are no less important. Reading the liver, in the adipose-driven majority of cases, as a major downstream site rather than the disease originator, while acknowledging its bidirectional feedback onto systemic metabolism, casts hepatic steatosis and its progression as an accessible signal of systemic metabolic health, a window onto the state of adipose tissue and fuel handling that is otherwise difficult to observe directly (41). It also clarifies why the quantity of hepatic fat is a weak guide to outcome while the biological context of its accumulation is decisive: the lipid is merely the visible deposit, whereas the injurious process reflects the severity of the underlying adipose and metabolic failure. For research, the framework directs attention towards the determinants of adipose dysfunction, the limits of subcutaneous expandability, the regulation of the inflammatory and adipokine response, and the basis of inter-individual susceptibility, as the variables most likely to explain why, among the many patients with type 2 diabetes and hepatic fat, only some progress to significant liver disease (53).

It should be emphasised that the adipose-centred framework developed here is explicitly phenotype-dependent: it is most directly applicable to the common phenotype of MASLD/MASH occurring in the context of obesity, visceral adiposity and type 2 diabetes, and is not proposed as universally applicable to all MASH phenotypes; it does not claim to account for the full clinical heterogeneity of the disease. A recognisable minority of patients develop MASLD without overt obesity or overt adipose dysfunction, so-called lean MASLD, in which genetic predisposition, particularly variants in PNPLA3 and TM6SF2, appears to play a proportionally larger role and adipose expandability failure a correspondingly smaller one. Other patients present with advanced fibrosis in the setting of comparatively modest metabolic derangement, a pattern that sits uneasily with a purely adipose-driven account and more plausibly reflects an intrinsically greater hepatic or fibrogenic susceptibility. These phenotypes should not be read as counterexamples that refute the systemic framework, but as a reminder that ATD is best understood as the dominant upstream lesion in the metabolically driven majority of cases, within a disease that remains mechanistically heterogeneous at its margins. Clarifying where a given patient sits along this spectrum, adipose-driven, genetically driven or an admixture of the two, is likely to be relevant both to prognosis and to the choice between systemically and hepatically directed therapy.

None of this denies the reality or the clinical weight of hepatic injury. The point is rather that the liver’s disease is best understood, studied and ultimately treated in the light of its systemic origin. A framework of this kind earns its place not by being proven but by being more faithful to the biology than the hepatocentric model it seeks to displace, and by directing enquiry and intervention towards the level at which the disorder actually begins. These recurring themes, adipokine dysregulation, insulin resistance and metabolic inflexibility, are best understood as facets of a single underlying lesion, dysfunctional adipose tissue, rather than as separate mechanisms. Several limitations of the conceptual framework itself should also be made explicit. Firstly, the model remains hypothesis-generating and predominantly inferential: it is largely derived from cross-sectional and mechanistic evidence, rather than from prospective studies designed to test the adipose-centred hypothesis against a hepatocentric alternative or against the other mechanistic models discussed above. Consequently, while the model is biologically coherent, it has not yet been prospectively validated, and its explanatory power remains inferential. Secondly, as discussed above, the framework applies most directly to the majority of MASLD/MASH associated with obesity and diabetes, and less directly to lean and genetically driven phenotypes. Thirdly, the framework should be read as identifying the most rational point of upstream intervention rather than as excluding a role for the other mechanisms, including gut-derived signals and intrinsic hepatocyte susceptibility, as the relative contribution of adipose dysfunction versus these other mechanisms cannot currently be quantified with precision.

This framework generates specific, testable predictions. Future prospective studies should assess whether quantitative measures of adipose dysfunction (for example, imaging-based indices of adipose expandability, circulating adipokine profiles or adipose-tissue insulin resistance indices) predict incident MASH, fibrosis progression or treatment response independently of hepatic fat content, BMI and conventional metabolic risk factors; whether interventions that improve adipose function produce hepatic benefit disproportionate to the degree of weight loss achieved; and whether the phenotypic distinction proposed here, adipose-driven versus genetically or intrinsically hepatic-driven disease, predicts differential response to systemically versus hepatically directed therapy.

## 8 Conclusions

MASH in type 2 diabetes has long been approached as a disease of the liver. The argument developed here is that it is more faithfully understood as the hepatic expression of a systemic disorder of adipose tissue. In the obesity- and visceral-adiposity-associated phenotype that predominates in type 2 diabetes, the causal narrative is continuous and biologically coherent: ATD renders adipose tissue immuno-inflammatory and endocrinologically deranged; a reconfigured adipokine secretome and sustained adipose inflammation impose and perpetuate insulin resistance; selective insulin resistance and the loss of metabolic flexibility convert a disorder of energy storage into a disorder of fuel handling; and the liver, by virtue of its portal exposure and metabolic centrality, becomes an organ on which the resulting lipid and inflammatory burden is heavily deposited and visibly expressed, while itself feeding back, through impaired insulin clearance, gluconeogenesis and hepatokine release, to worsen the systemic insulin resistance that sustains the cycle. ATD is presented as one major upstream mechanism within a heterogeneous, multi-organ and bidirectional disease process, rather than as the universal origin of MASH. Hepatic steatosis is the predictable consequence of failed peripheral lipid buffering, and steatohepatitis the readout of lipotoxic, immunometabolic injury rather than an autonomous hepatic process. As throughout this review, the adipose-centred model advanced here should be understood as a conceptual framework for the pathogenesis of MASLD/MASH, and not as an alternative to the other established pathogenic mechanisms discussed above.

The principal contribution of this reframing is to relocate the disorder’s centre of gravity. By identifying the liver, in the adipose-driven majority of cases, as a major downstream site of systemic metabolic injury rather than the primary disease originator, while recognising its capacity to feed back into and amplify systemic dysfunction, it directs both mechanistic enquiry and the most rational point of therapeutic intervention upstream, to adipose biology and the regulation of fuel handling, and it offers a unifying explanation for observations, the weak prognostic value of hepatic fat, the systemic determinants of progression, the efficacy of interventions that act on adipose tissue, that a purely hepatic account accommodates only with difficulty. We advance the model as an interpretative framework rather than a settled conclusion, and as a foundation for the more detailed mechanistic and clinical questions that follow from taking the systemic origin of MASH seriously. The fatty liver of the patient with type 2 diabetes, on this reading, is rarely just a fatty liver; it is the visible sign of a disorder that begins in the adipose organ.

## References

[1] Rinella ME Lazarus JV Ratziu V Francque SM Sanyal AJ Kanwal F . A multisociety Delphi consensus statement on new fatty liver disease nomenclature. Hepatology. (2023) 78:1966–86. doi: 10.1097/HEP.0000000000000520 37363821 PMC10653297

[2] Younossi ZM Golabi P Price JK Owrangi S Gundu-Rao N Satchi R . The global epidemiology of nonalcoholic fatty liver disease and nonalcoholic steatohepatitis among patients with type 2 diabetes. Clin Gastroenterol Hepatol. (2024) 22:1999–2010.e8. doi: 10.1016/j.cgh.2024.03.006 38521116

[3] Stefan N Yki-Järvinen H Neuschwander-Tetri BA . Metabolic dysfunction-associated steatotic liver disease: heterogeneous pathomechanisms and effectiveness of metabolism-based treatment. Lancet Diabetes Endocrinol. (2025) 13:134–48. doi: 10.1016/S2213-8587(24)00318-8 39681121

[4] Byrne CD Targher G . NAFLD: a multisystem disease. J Hepatol. (2015) 62:S47–64. doi: 10.1016/j.jhep.2014.12.012 25920090

[5] Bays H Abate N Chandalia M . Adiposopathy: sick fat causes high blood sugar, high blood pressure and dyslipidemia. Future Cardiol. (2005) 1:39–59. doi: 10.1517/14796678.1.1.39 19804060

[6] Bays H . Adiposopathy, "sick fat," Ockham's razor, and resolution of the obesity paradox. Curr Atheroscler Rep. (2014) 16:409. doi: 10.1007/s11883-014-0409-1 24659222 PMC3972445

[7] Virtue S Vidal-Puig A . Adipose tissue expandability, lipotoxicity and the Metabolic Syndrome--an allostatic perspective. Biochim Biophys Acta. (2010) 1801:338–49. doi: 10.1016/j.bbalip.2009.12.006 20056169

[8] Tchernof A Després JP . Pathophysiology of human visceral obesity: an update. Physiol Rev. (2013) 93:359–404. doi: 10.1152/physrev.00033.2011 23303913

[9] Ouchi N Parker JL Lugus JJ Walsh K . Adipokines in inflammation and metabolic disease. Nat Rev Immunol. (2011) 11:85–97. doi: 10.1038/nri2921 21252989 PMC3518031

[10] Zhang Y Sowers JR Ren J . Targeting autophagy in obesity: from pathophysiology to management. Nat Rev Endocrinol. (2018) 14:356–76. doi: 10.1038/s41574-018-0009-1 29686432

[11] Gastaldelli A Cusi K . From NASH to diabetes and from diabetes to NASH: mechanisms and treatment options. JHEP Rep. (2019) 1:312–28. doi: 10.1016/j.jhepr.2019.07.002 32039382 PMC7001557

[12] Goodpaster BH Bergman BC Brennan AM Sparks LM . Intermuscular adipose tissue in metabolic disease. Nat Rev Endocrinol. (2023) 19:285–98. doi: 10.1038/s41574-022-00784-2 36564490

[13] Pujia R Tarsitano MG Arturi F De Lorenzo A Lenzi A Pujia A . Advances in phenotyping obesity and in its dietary and pharmacological treatment: a narrative review. Front Nutr. (2022) 9:804719. doi: 10.3389/fnut.2022.804719 35242796 PMC8885626

[14] Hall KD Ayuketah A Brychta R Cai H Cassimatis T Chen KY . Ultra-processed diets cause excess calorie intake and weight gain: an inpatient randomized controlled trial of ad libitum food intake. Cell Metab. (2019) 30:67–77.e3. doi: 10.1016/j.cmet.2019.05.008 31105044 PMC7946062

[15] Geladari EV Kounatidis D Christodoulatos GS Psallida S Pavlou A Geladari CV . Ultra-processed foods and metabolic dysfunction-associated steatotic liver disease (MASLD): what is the evidence so far? Nutrients. (2025) 17:2098. doi: 10.3390/nu17132098 40647203 PMC12251232

[16] Weisberg SP McCann D Desai M Rosenbaum M Leibel RL Ferrante AW Jr . Obesity is associated with macrophage accumulation in adipose tissue. J Clin Invest. (2003) 112:1796–808. doi: 10.1172/JCI19246 14679176 PMC296995

[17] Xu H Barnes GT Yang Q Tan G Yang D Chou CJ . Chronic inflammation in fat plays a crucial role in the development of obesity-related insulin resistance. J Clin Invest. (2003) 112:1821–30. doi: 10.1172/JCI19451 14679177 PMC296998

[18] Lumeng CN Bodzin JL Saltiel AR . Obesity induces a phenotypic switch in adipose tissue macrophage polarization. J Clin Invest. (2007) 117:175–84. doi: 10.1172/JCI29881 17200717 PMC1716210

[19] Kanda H Tateya S Tamori Y Kotani K Hiasa K Kitazawa R . MCP-1 contributes to macrophage infiltration into adipose tissue, insulin resistance, and hepatic steatosis in obesity. J Clin Invest. (2006) 116:1494–505. doi: 10.1172/JCI26498 16691291 PMC1459069

[20] Hotamisligil GS . Inflammation and metabolic disorders. Nature. (2006) 444:860–7. doi: 10.1038/nature05485 17167474

[21] Yao J Wu D Qiu Y . Adipose tissue macrophage in obesity-associated metabolic diseases. Front Immunol. (2022) 13:977485. doi: 10.3389/fimmu.2022.977485 36119080 PMC9478335

[22] Schmid A Karrasch T Schäffler A . The emerging role of bile acids in white adipose tissue. Trends Endocrinol Metab. (2023) 34:718–34. doi: 10.1016/j.tem.2023.08.002 37648561

[23] Shimobayashi M Albert V Woelnerhanssen B Frei IC Weissenberger D Meyer-Gerspach AC . Insulin resistance causes inflammation in adipose tissue. J Clin Invest. (2018) 128:1538–50. doi: 10.1172/JCI96139 29528335 PMC5873875

[24] Kadowaki T Yamauchi T Kubota N Hara K Ueki K Tobe K . Adiponectin and adiponectin receptors in insulin resistance, diabetes, and the metabolic syndrome. J Clin Invest. (2006) 116:1784–92. doi: 10.1172/JCI29126 16823476 PMC1483172

[25] Stern JH Rutkowski JM Scherer PE . Adiponectin, leptin, and fatty acids in the maintenance of metabolic homeostasis through adipose tissue crosstalk. Cell Metab. (2016) 23:770–84. doi: 10.1016/j.cmet.2016.04.011 27166942 PMC4864949

[26] Steppan CM Bailey ST Bhat S Brown EJ Banerjee RR Wright CM . The hormone resistin links obesity to diabetes. Nature. (2001) 409:307–12. doi: 10.1038/35053000 11201732

[27] Accili D Deng Z Liu Q . Insulin resistance in type 2 diabetes mellitus. Nat Rev Endocrinol. (2025) 21:413–26. doi: 10.1038/s41574-025-01114-y 40247011

[28] Brown MS Goldstein JL . Selective versus total insulin resistance: a pathogenic paradox. Cell Metab. (2008) 7:95–6. doi: 10.1016/j.cmet.2007.12.009 18249166

[29] Samuel VT Shulman GI . Mechanisms for insulin resistance: common threads and missing links. Cell. (2012) 148:852–71. doi: 10.1016/j.cell.2012.02.017 22385956 PMC3294420

[30] Randle PJ Garland PB Hales CN Newsholme EA . The glucose fatty-acid cycle. Its role in insulin sensitivity and the metabolic disturbances of diabetes mellitus. Lancet. (1963) 1:785–9. doi: 10.1016/s0140-6736(63)91500-9 13990765

[31] Kelley DE Mandarino LJ . Fuel selection in human skeletal muscle in insulin resistance: a reexamination. Diabetes. (2000) 49:677–83. doi: 10.2337/diabetes.49.5.677 10905472

[32] Goodpaster BH Sparks LM . Metabolic flexibility in health and disease. Cell Metab. (2017) 25:1027–36. doi: 10.1016/j.cmet.2017.04.015 28467922 PMC5513193

[33] Galgani JE Moro C Ravussin E . Metabolic flexibility and insulin resistance. Am J Physiol Endocrinol Metab. (2008) 295:E1009–17. doi: 10.1152/ajpendo.90558.2008 18765680 PMC2584808

[34] Dash D Koiri RK . Metabolic dysfunction associated steatotic liver disease as a disorder of hepatic bioenergetic timing. In: Medical Hypotheses. (Amsterdam: Elsevier) (2026). 2111:11970. doi: 10.1016/j.mehy.2026.111970

[35] Rui L . Energy metabolism in the liver. Compr Physiol. (2014) 4:177–97. doi: 10.1002/cphy.c130024 24692138 PMC4050641

[36] Donnelly KL Smith CI Schwarzenberg SJ Jessurun J Boldt MD Parks EJ . Sources of fatty acids stored in liver and secreted via lipoproteins in patients with nonalcoholic fatty liver disease. J Clin Invest. (2005) 115:1343–51. doi: 10.1172/JCI23621 15864352 PMC1087172

[37] Chaurasia B Summers SA . Ceramides. Lipotoxic inducers of metabolic disorders. Trends Endocrinol Metab. (2015) 26:538–50. doi: 10.1016/j.tem.2015.07.006 26412155

[38] Neuschwander-Tetri BA . Hepatic lipotoxicity and the pathogenesis of nonalcoholic steatohepatitis: the central role of nontriglyceride fatty acid metabolites. Hepatology. (2010) 52:774–88. doi: 10.1002/hep.23719 20683968

[39] Fromenty B Roden M . Mitochondrial alterations in fatty liver diseases. J Hepatol. (2023) 78:415–29. doi: 10.1016/j.jhep.2022.09.020 36209983

[40] Ozcan U Cao Q Yilmaz E Lee AH Iwakoshi NN Ozdelen E . Endoplasmic reticulum stress links obesity, insulin action, and type 2 diabetes. Science. (2004) 306:457–61. doi: 10.1126/science.1103160 15486293

[41] Targher G Corey KE Byrne CD Roden M . The complex link between NAFLD and type 2 diabetes mellitus - mechanisms and treatments. Nat Rev Gastroenterol Hepatol. (2021) 18:599–612. doi: 10.1038/s41575-021-00448-y 33972770

[42] Manshi Dash D Koiri RK . The bidirectional relationship between diabetes mellitus and liver disease: mechanisms, clinical implications, and management strategies. Endocrine. (2026) 91:74. doi: 10.1007/s12020-026-04561-3 41697495

[43] Tilg H Moschen AR . Evolution of inflammation in nonalcoholic fatty liver disease: the multiple parallel hits hypothesis. Hepatology. (2010) 52:1836–46. doi: 10.1002/hep.24001 21038418

[44] Szabo G Csak T . Inflammasomes in liver diseases. J Hepatol. (2012) 57:642–54. doi: 10.1016/j.jhep.2012.03.035 22634126

[45] Kanaoujiya R Dash D Koiri RK Mustafa G Chauhan PK Bartaria D . Synthesis, characterisation, computational study, amelioration of ruthenium kesar nanoparticle, antioxidant and glycolytic enzyme activity alterations in cirrhotic liver extract. In: Sci Rep. (London: Springer Nature). (2026). 16:13714. doi: 10.1038/s41598-026-41812-9 PMC13125602 41839925

[46] Tilg H Adolph TE Trauner M . Gut-liver axis: pathophysiological concepts and clinical implications. Cell Metab. (2022) 34:1700–18. doi: 10.1016/j.cmet.2022.09.017 36208625

[47] Friedman SL Neuschwander-Tetri BA Rinella M Sanyal AJ . Mechanisms of NAFLD development and therapeutic strategies. Nat Med. (2018) 24:908–22. doi: 10.1038/s41591-018-0104-9 29967350 PMC6553468

[48] Manothiya P Dash D Koiri RK . Gut microbiota dysbiosis and the gut-liver-brain axis: mechanistic insights into hepatic encephalopathy. In: Medicine in Microecology. (Amsterdam: Elsevier) (2025). 26:100157. doi: 10.1016/j.medmic.2025.100157

[49] Rajak M Dash D Koiri RK . Microbiota-derived metabolites in cancer: dual roles in pathogenesis and opportunities for therapy. In: Medicine in Drug Discovery. (Amsterdam: Elsevier) (2026). 29:100245. doi: 10.1016/j.medidd.2025.100245

[50] Cusi K Orsak B Bril F Lomonaco R Hecht J Ortiz-Lopez C . Long-term pioglitazone treatment for patients with nonalcoholic steatohepatitis and prediabetes or type 2 diabetes mellitus: a randomized trial. Ann Intern Med. (2016) 165:305–15. doi: 10.7326/M15-1774 27322798

[51] Sanyal AJ Newsome PN Kliers I Østergaard LH Long MT Kjær MS . Phase 3 trial of semaglutide in metabolic dysfunction-associated steatohepatitis. N Engl J Med. (2025) 392:2089–99. doi: 10.1056/NEJMoa2413258 40305708

[52] Lassailly G Caiazzo R Ntandja-Wandji LC Gnemmi V Baud G Verkindt H . Bariatric surgery provides long-term resolution of nonalcoholic steatohepatitis and regression of fibrosis. Gastroenterology. (2020) 159:1290–1301.e5. doi: 10.1053/j.gastro.2020.06.006 32553765

[53] Younossi ZM Golabi P de Avila L Paik JM Srishord M Fukui N . The global epidemiology of NAFLD and NASH in patients with type 2 diabetes: A systematic review and meta-analysis. J Hepatol. (2019) 71:793–801. doi: 10.1016/j.jhep.2019.06.021 31279902

